# *Physalis peruviana*-Derived Physapruin A (PHA) Inhibits Breast Cancer Cell Proliferation and Induces Oxidative-Stress-Mediated Apoptosis and DNA Damage

**DOI:** 10.3390/antiox10030393

**Published:** 2021-03-05

**Authors:** Tzu-Jung Yu, Yuan-Bin Cheng, Li-Ching Lin, Yi-Hong Tsai, Bo-Yi Yao, Jen-Yang Tang, Fang-Rong Chang, Chia-Hung Yen, Fu Ou-Yang, Hsueh-Wei Chang

**Affiliations:** 1 Graduate Institute of Natural Products, Kaohsiung Medical University, Kaohsiung 80708, Taiwan; u109831002@kmu.edu.tw (T.-J.Y.); r960134@kmu.edu.tw (Y.-H.T.); u106531009@kmu.edu.tw (B.-Y.Y.); aaronfrc@kmu.edu.tw (F.-R.C.); chyen@kmu.edu.tw (C.-H.Y.); 2 Department of Marine Biotechnology and Resources, National Sun Yat-sen University, Kaohsiung 80424, Taiwan; jmb@mail.nsysu.edu.tw; 3 Department of Radiation Oncology, Chi-Mei Foundation Medical Center, Tainan 71004, Taiwan; 8508a6@mail.chimei.org.tw; 4 School of Medicine, Taipei Medical University, Taipei 11031, Taiwan; 5 Chung Hwa University Medical Technology, Tainan 71703, Taiwan; 6 School of Post-Baccalaureate Medicine, Kaohsiung Medical University, Kaohsiung 80708, Taiwan; reyata@kmu.edu.tw; 7 Department of Radiation Oncology, Kaohsiung Medical University Hospital, Kaohsiung 80708, Taiwan; 8 Division of Breast Surgery, Department of Surgery, Kaohsiung Medical University Hospital, Kaohsiung 80708, Taiwan; 9 Center for Cancer Research, Kaohsiung Medical University, Kaohsiung 80708, Taiwan; 10 Cancer Center, Kaohsiung Medical University Hospital, Kaohsiung 80708, Taiwan; 11 Institute of Medical Science and Technology, National Sun Yat-sen University, Kaohsiung 80424, Taiwan; 12 Department of Medical Research, Kaohsiung Medical University Hospital, Kaohsiung 80708, Taiwan; 13 Department of Biomedical Science and Environmental Biology, Ph.D. Program in Life Sciences, College of Life Sciences, Kaohsiung Medical University, Kaohsiung 80708, Taiwan

**Keywords:** withanolide, breast cancer, apoptosis, oxidative stress, DNA damage

## Abstract

Breast cancer expresses clinically heterogeneous characteristics and requires multipurpose drug development for curing the different tumor subtypes. Many withanolides have been isolated from *Physalis* species showing anticancer effects, but the anticancer function of physapruin A (PHA) has rarely been investigated. In this study, the anticancer properties of PHA in breast cancer cells were examined by concentration and time-course experiments. In terms of cellular ATP content, PHA inhibited the proliferation of three kinds of breast cancer cells: MCF7 (estrogen receptor (ER)+, progesterone receptor (PR)+/−, human epidermal growth factor receptor 2 (HER2)−), SKBR3 (ER−/PR−/HER2+), and MDA-MB-231 (triple-negative). Moreover, PHA induced G2/M arrest in MCF7 and MDA-MB-231 cells. In terms of flow cytometry, PHA induced the generation of reactive oxygen species (ROS), the generation of mitochondrial superoxide, mitochondrial membrane potential depletion, and γH2AX-detected DNA damage in breast cancer MCF7 and MDA-MB-231 cells, which were suppressed by the ROS inhibitor *N*-acetylcysteine (NAC). In terms of flow cytometry and Western blotting, PHA induced apoptotic expression (annexin V, and intrinsic and extrinsic apoptotic signaling), which was suppressed by NAC and an apoptosis inhibitor (Z-VAD-FMK), in breast cancer cells. Therefore, PHA is a potential anti-breast-cancer natural product that modulates the oxidative-stress response, cell-cycle disturbance, apoptosis, and γH2AX-detected DNA damage.

## 1. Introduction

Breast cancer was the top female cancer type for the recorded new cancer cases in Taiwan [1] and the United States [2] in 2020. Breast cancer expresses clinically heterogeneous characteristics that show differential expression for estrogen receptor (ER), human epidermal growth factor receptor 2 (HER2), and progesterone receptor (PR) [3]. For breast cancer treatments, targeted therapies for ER-positive [4], HER2-positive [5], and PR-positive [6] subtypes have been well reviewed. However, the subtype triple-negative breast cancer (TNBC) [3,7] is characterized by a lack of ER, HER2, and PR expression and, therefore, shows no response during therapy targeted towards these receptors.

Chemotherapy provides an alternative strategy for curing all subtypes of breast cancer cells. However, different subtypes have different therapeutic responses to chemotherapy, which may partly be explained by the finding that different subtypes show different combinations of negative and positive expression for ER, HER2, and PR [8]. Chemotherapy for curing breast cancer is occasionally associated with side effects [9] or drug resistance [10]. Accordingly, drug development for breast cancer therapy remains a challenge.

*Physalis peruviana* L. belongs to the family Solanaceae, containing at least 120 species [11]. Many *Physalis* species are used for traditional medicinal applications in Asia and South America [12] since they contain the bioactive compound class withanolides. Withanolides include more than 300 natural C-28 steroidal lactones [12], and some exhibit anticancer functions [13,14,15,16,17,18,19,20]. For example, withaferin A [17,19], 4β-hydroxywithanolide [18,19], and withanone [13] have demonstrated anti-breast-cancer effects. However, the anticancer effects of several kinds of withanolides have not been fully explored. The further identification of the anticancer effects of withanolides is warranted.

Although several withanolides are reported in anticancer studies, this does not hold for physapruin A (PHA), which was firstly isolated from *Physalis peruviana* [21] in 1993. Recently, PHA was shown to demonstrate antiproliferative abilities for prostate (LNCaP) and renal (ACHN) cancer cells [22]. However, anticancer functions for other types of cancer cells have rarely been reported. Particularly, the detailed anticancer mechanisms of PHA have not been investigated for breast cancer cells yet. Herein, we investigated its antiproliferative effects and explored its mechanisms of action for the case of PHA-treated breast cancer cells.

## 2. Materials and Methods

### 2.1. Plant Material, Extraction, and Compound Isolation

Specimens of *Physalis peruviana* and their roots were harvested in Chiayi county, Taiwan, in July 2017. Prof. Yuan-Bin Cheng recognized the specimens (no. KMU-ppr1), and the specimens were stored in the Department of Marine Biotechnology and Resources, National Sun Yat-sen University, Kaohsiung.

The roots of *P. peruviana* (20.0 kg) were extracted by ethanol thrice. After removing the organic solvent, a crude extract (361.9 g) was obtained. The crude extract was divided into EtOAc-soluble and H_2_O-soluble portions (45.2 g). The EtOAc-soluble layer was then divided into hexane-soluble and 75% EtOH-soluble portions. The 75% EtOH-soluble portion (116.0 g) was separated by a silica gel flash column (hexane–EtOAc–MeOH, 2:1:0 to 0:0:1) to produce eight fractions (Fr.1–Fr.8). Fr.5 (20.4 g) was separated on a Si gel open column, eluted with CH_2_Cl_2_–MeOH (40:1 to 0:1) to yield a triterpenoid-enriched fraction F5A–F5F. F5C (4.1 g) was subjected to an RP-C_18_ column, eluted with H_2_O–MeOH to provide subfractions F5C1–F5C8. F5C4 (3.4 g) was purified by a silica gel column, stepwise eluted with hexane–acetone–MeOH (4:1:0 to 0:0:1), yielding subfractions F5C4A–F5C4E. F5C4A (1.8 g) was re-separated by a silica gel column, stepwise eluted with hexane–acetone–MeOH (4:1:0 to 0:0:1) to yield fractions F5C4A1–F5C4A8. Fraction F5C4A7 (587.3 mg) was further isolated by a silica gel column, stepwise eluted with CH_2_Cl_2_–EtOAc–MeOH (3:1:0 to 0:0:1) to afford fractions F5C4A7A–F5C4A7F. F5C4A7D (287.1 mg) was purified by a silica gel column, stepwise eluted with CH_2_Cl_2_–MeOH (35:1 to 0:1) to obtain fraction F5C4A7D6 (83.8 mg). This fraction was finally purified by NP–HPLC (Phenomenex CN; 10 × 250 mm; flow rate, 2.0 mL/min; *n*-hexane–EtOAc–MeOH, 15:10:1) to provide the PHA compound (26.5 mg), which was further investigated here. The purity of each sample (>99%) was verified by analytical HPLC before the bioassays.

### 2.2. PHA Chemical Profile

PHA was obtained as a white solid and identified based on its electrospray ionization mass spectrometer (ESI MS) and NMR characteristics. The molecular formula of PHA was determined to be C_28_H_38_O_7_ based on the ESIMS spectrum, which showed a parent ion peak at *m*/*z* 509 [M+Na]^+^. The IR spectrum revealed the presence of hydroxy (3367 cm^−1^) and carbonyl (1647 and 1719 cm^−1^) functionalities. The proton NMR spectrum of PHA revealed five methyl singlets (δ_H_ 1.94, 1.87, 1.44, 1.41, and 1.12), three olefinic methines (δ_H_ 6.77 dd, *J* = 10.0, 4.6 Hz; δ_H_ 5.94 m; δ_H_ 5.93 d, *J* = 10.0 Hz), and two oxymethines (δ_H_ 4.94 dd, *J* = 10.8, 4.6 Hz and δ_H_ 4.62 d, *J* = 4.6 Hz). In the ^13^C-NMR and DEPT spectra, 28 carbon signals were observed, consisting of one ketone (δ_C_ 204.2), one ester carbonyl (δ_C_ 166.8), three olefinic methines (δ_C_ 146.1, 130.4, and 128.6), three olefinic nonprotonated carbons (δ_C_ 150.8, 139.4, and 121.5), two oxymethines (δ_C_ 81.7 and 69.2), three oxygen-bearing quaternary carbons (δ_C_ 88.5, 81.7, and 79.4), and five methyls (δ_C_ 22.6, 21.3, 20.2, 19.7, and 12.6). On the basis of the above data, the structure of PHA was confirmed.

### 2.3. Reagents and Antibody Information

Pretreatment with an oxidative-stress inhibitor, 10 mM *N*-acetylcysteine (NAC) (Sigma-Aldrich; St. Louis, MO, USA), for 1 h [23,24] was applied to confirm the involvement of oxidative stress in the PHA post-treatment experiments. For the drug treatments, PHA stock (20 mM) was made in dimethyl sulfoxide (DMSO). An NAC stock (400 mM) was made in double-distilled water. The apoptosis inhibitor Z-VAD-FMK (ZVAD) (Selleckchem.com; Houston, TX, USA) was made in DMSO. In the Western blotting analysis, cleaved form types of primary antibodies (1:1000) for poly (ADP-ribose) polymerase (c-PARP) and caspases 3, 8, and 9 (c-Cas 3, 8, and 9) (Cell Signaling Technology; Danvers, MA, USA) as well as a control primary antibody (1:5000) for β-actin (Sigma-Aldrich) were chosen [25]. A p-Histone H2A.X (γH2AX) primary antibody (Santa Cruz Biotechnology; Santa Cruz, CA, USA) and secondary antibody labeled with Alexa 488 (Cell Signaling Technology) were used for the flow cytometry experiments.

### 2.4. Cell Culture and ATP Assay

Three ATCC (Manassas, VA, USA) human breast cancer cell lines (SKBR3, MCF7, and MDA-MB-231) were chosen. These cells were grown with a mixed medium (Dulbecco’s Modified Eagle Medium (DMEM) and F12) formulated in the ratio of 3:2 and supplemented with 10% bovine serum and cell culture antibiotics (Gibco, Grand Island, NY, USA) for common cell culture use (otherwise at 5% CO_2_ and 37 °C). The cell viability analysis was performed using an ATP commercial kit (PerkinElmer Life Sciences, Boston, MA, USA) [26].

### 2.5. Cell-Cycle Assay

Cells were processed with 1 μg/mL of the DNA dye 7-aminoactinomycin D (7AAD) (Biotium Inc., Hayward, CA, USA) for 30 min of treatment at 37 °C for the cell-cycle assay as described previously [15]. The cell-cycle assay was performed using a flow cytometer (Guava^®^ easyCyte^TM^; Luminex, TX, USA). Each cell-cycle phase was analyzed using the FlowJo tool (Becton-Dickinson; Franklin Lakes, NJ, USA).

### 2.6. Annexin V/7AAD Dual Staining for Apoptosis and Necrosis Detection

Cells were harvested, washed, and mixed with an annexin V/7AAD dual staining kit (Strong Biotech Corp., Taipei, Taiwan) for the apoptosis assay as described previously [27] using flow cytometry (Guava^®^ easyCyte^TM^) and the FlowJo software (Becton-Dickinson). The 7AAD (+/−)/annexin V (+) (%) ratio is regarded as the apoptosis (%), and the 7AAD (+)/annexin V (−) (%) ratio is regarded as the necrosis (%) [28,29].

### 2.7. ROS Detection

Cells were processed with 10 μM 2′,7′-dichlorodihydrofluorescein diacetate (H_2_DCF-DA) dye (Sigma-Aldrich) for 30 min of treatment at 37 °C in the ROS assay as described previously [16] using flow cytometry (Guava^®^ easyCyte^TM^). The ROS intensity was analyzed using the FlowJo tool.

### 2.8. Mitochondrial Superoxide (MitoSOX) Detection

Cells were processed with 50 nM MitoSOX™ Red dye (Thermo Fisher Scientific, Carlsbad, CA, USA) for 30 min of treatment at 37 °C for the MitoSOX assay as described previously [30] using flow cytometry (Guava^®^ easyCyte^TM^). The MitoSOX intensity was analyzed using the FlowJo tool.

### 2.9. Mitochondrial Membrane Potential (MitoMP) Detection

Cells were processed with 5 nM MitoProbe^TM^ DiOC_2_(3) (Thermo Fisher Scientific, Carlsbad, CA, USA) for 20 min of treatment at 37 °C for the MitoMP assay as described previously [31] using flow cytometry (Guava^®^ easyCyte^TM^) and the FlowJo tool (Becton-Dickinson).

### 2.10. Quantitative RT-PCR

The total RNA extraction, cDNA reverse transcription, and quantitative RT-PCR as well as the primer information for the glutathione-disulfide reductase (*GSR*) and *GAPDH* genes were described previously [32]. *GSR* mRNA expression is presented as fold activation (log_2_ scale) relative to the control, GAPDH.

### 2.11. γ. H2AX DNA-Damage Detection

The γH2AX primary antibody (1:500; 1 h, 4 °C) and Alexa 488-modified secondary antibody incubation (30 min, 37 °C), and 5 μg/mL of 7AAD staining (30 min, 37 °C) were performed for the γH2AX assay as previously described [33] using flow cytometry (Guava^®^ easyCyte^TM^) and the FlowJo tool (Becton-Dickinson).

### 2.12. Statistical Analysis

Statistical analysis was performed by using the analysis of variance (ANOVA) associated with the JMP^®^12-based HSD post-hoc test when the multiple comparisons were considered.

## 3. Results

### 3.1. PHA Provided Antiproliferative Effects for Breast Cancer Depending on ROS

**Figure 1:**
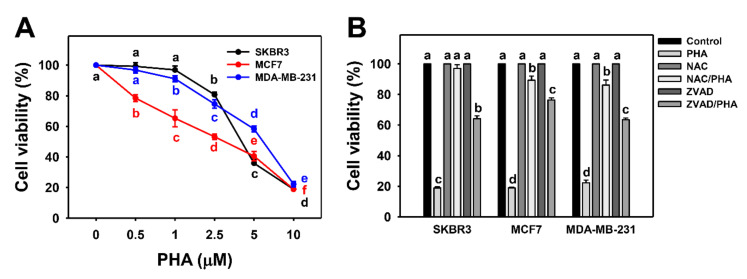
Physapruin (PHA) decreased ATP- detected cell viabilities of breast cancer cells. (**A**) Concentration effect of PHA in an ATP assay. As indicated, three breast cancer cell lines were incubated with 0 (0.05% DMSO as control), 0.5, 1, 2.5, 5, and 10 μM PHA for 24 h. (**B**) NAC or ZVAD pretreatment responses in ATP changes in PHA-treated breast cancer cells. Cells pretreated with either NAC (10 mM for 1 h) or ZVAD (100 μM for 2 h) and post-treated with 0 (control with 0.05% DMSO) and 10 μM PHA for 24 h, i.e., NAC/PHA or ZVAD/PHA. Data, means ± SDs (*n* = 3 independent experiments). Experiments without the same top-labeled letters indicate significant changes according to a multiple ANOVA comparison (*p* < 0.05 to 0.0001).

In an ATP assay for 24 h, the cell viability of breast cancer (SKBR3, MCF7, and MDA-MB-231) cells was inhibited by PHA (Figure 1A). Pretreatments with NAC or ZVAD could mostly or moderately suppress the PHA-induced antiproliferative effects against the three types of breast cancer cells (Figure 1B).

### 3.2. PHA Changed Cell-Cycle Progression of Breast Cancer Cells

**Figure 2:**
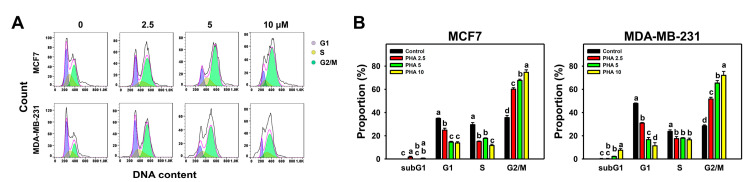
PHA redistributed the cell-cycle phases of two breast cancer cell lines. (**A**,**B**) Cell-cycle phase patterns and statistical analysis for concentration responses to PHA. Cells were incubated with PHA (0 (0.05% DMSO as control), 2.5, 5, and 10 μM) for 24 h, i.e., control, PHA 2.5, PHA 5, and PHA 10. Data, means ± SDs (*n* = 3 independent experiments). Experiments without the same top-labeled letters indicate significant changes according to multiple ANOVA comparisons (*p* < 0.05 to 0.0001).

The variations in the cell-cycle phases in the breast cancer cells following PHA treatment were detected by flow cytometric analysis (Figure 2A). Different PHA concentrations decreased the G1 population, decreased the S population, and increased G2/M arrest in breast cancer cells, although the subG1 population was small (Figure 2B).

### 3.3. PHA Triggered More Apoptosis Than Necrosis in Breast Cancer Cells

**Figure 3:**
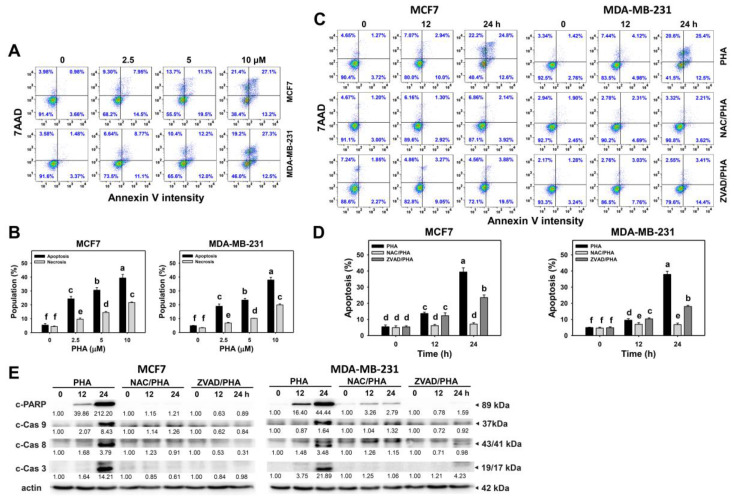
PHA triggers apoptosis and activates caspases in breast cancer cells. (**A**,**B**) Annexin V/7AAD patterns and statistical analysis for concentration responses to PHA. Cells were treated with PHA (0 (0.05% DMSO as control), 2.5, 5, and 10 μM) for 24 h. 7AAD (+/−)/annexin V (+) (%) ratio is regarded as the apoptosis (%), and 7AAD (+)/annexin V (−) (%) ratio is regarded as the necrosis (%) [28,29]. (**C**,**D**) Pattern and statistical analysis for NAC or ZVAD pretreatment responses in annexin V changes in PHA-post-treated breast cancer cells. Following pretreatment with either NAC (10 mM for 1 h) or ZVAD (100 μM for 2 h), cells were post-treated with 0 (0.05% DMSO as control) and 10 μM PHA for 0, 12, and 24 h, i.e., NAC/PHA or ZVAD/PHA. Data, means ± SDs (*n* = 3 independent experiments). Experiments without the same top-labeled letters indicate significant changes based on multiple comparisons (*p* < 0.05 to 0.0001). (**E**) Apoptosis-signaling expression of PHA-treated breast cancer cells. Cleaved PARP (c-PARP) and cleaved caspase 3, 8, and 9 (c-Cas 3, 8, and 9) expression is compared with reference to β-actin expression.

The changes in the annexin V/7AAD patterns at different concentrations and exposure times of PHA-treated breast cancer cells were detected by flow cytometry (Figure 3A,C). PHA induced concentration- and time-dependent increases in apoptosis (annexin V (+)) (%) in the breast cancer cells (MCF7 and MDA-MB-231) (Figure 3B,D). For Western blotting, the apoptosis expression was further examined at different time intervals. Apoptosis-signaling proteins such as c-PAPR, c-Cas 9, c-Cas 8, and c-Cas 3 were mildly increased at 12 h and dramatically increased at 24 h of PHA treatment in the breast cancer cells (Figure 3E). Moreover, annexin V (−)/7AAD (+)-defined necrosis [28,29] in the breast cancer cells was induced by PHA (Figure 3B). PHA induced more apoptosis than necrosis in breast cancer cells.

To validate the role of ROS in the apoptosis induction, NAC was used to pretreat the PHA-treated breast cancer cells. The NAC pretreatment suppressed the PHA-induced apoptosis (annexin V) in the breast cancer cells at 12 and 24 h (Figure 3D). The NAC pretreatment suppressed the PHA-induced overexpression of apoptosis-signaling proteins in the breast cancer cells (Figure 3E). Moreover, the PHA-induced apoptosis (annexin V) was suppressed by the apoptosis inhibitor ZVAD at 24 h (Figure 3D). The PHA-induced overexpression of apoptosis-signaling proteins was suppressed by ZVAD (Figure 3E), confirming the apoptosis induction by PHA.

### 3.4. PHA Substantially Upregulated ROS Generation in Breast Cancer Cells

**Figure 4:**
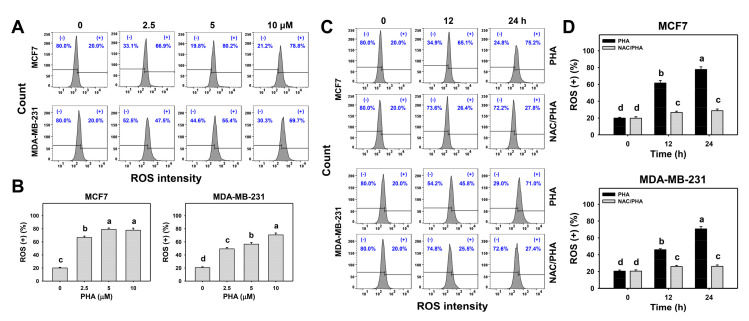
PHA triggered ROS generation in two breast cancer cell lines. (**A**,**B**) ROS patterns and statistical analysis for concentration responses to PHA. Cells were incubated with PHA (0 (0.05% DMSO as control), 2.5, 5, and 10 μM) for 24 h. (+) indicates ROS (+) (%). (**C**,**D**) Pattern and statistical analysis for NAC pretreatment responses in ROS intensity changes in PHA-post-treated breast cancer cells. Following pretreatment with NAC, cells were processed with post-treatment with 0 (control with 0.05% DMSO) and 10 μM PHA for 0, 12, and 24 h, i.e., PHA and NAC/PHA. Data, means ± SDs (*n* = 3 independent experiments). Experiments without the same top-labeled letters indicate significant changes according to multiple ANOVA comparison (*p* < 0.05 to 0.0001).

The changes in the ROS generation patterns with different concentrations and exposure times of PHA-treated breast cancer cells were detected by flow cytometry (Figure 4A,C). PHA induced concentration- and time-course-dependent increases in the ROS (+) (%) in the breast cancer cells (Figure 4B,D).

To validate the function of ROS, NAC was used to pretreat PHA-treated breast cancer cells. The NAC pretreatment suppressed the PHA-induced ROS overexpression in the breast cancer cells (Figure 4D).

### 3.5. PHA Substantially Upregulated MitoSOX in Breast Cancer Cells

**Figure 5:**
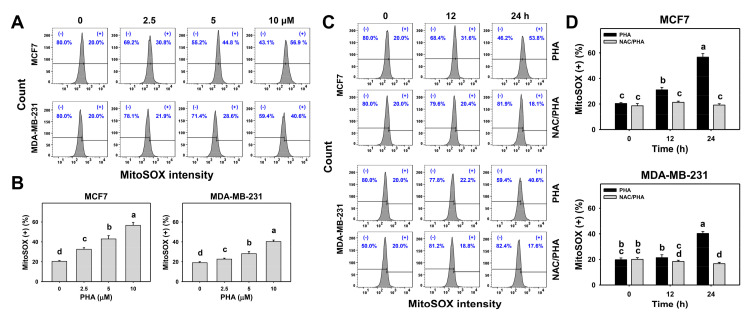
PHA triggered MitoSOX generation in two breast cancer cell lines. (**A**,**B**) MitoSOX patterns and statistical analysis for concentration responses to PHA. Cells were incubated with PHA (0 (0.05% DMSO as control), 2.5, 5, and 10 μM) for 24 h. (+) indicates MitoSOX (+) (%). (**C**,**D**) Pattern and statistical analysis for NAC pretreatment responses in MitoSOX intensity changes in PHA-post-treated breast cancer cells. Following pretreatment with NAC, cells were processed with post-treatment for 0 (control with 0.05% DMSO) and 10 μM PHA for 0, 12, and 24 h, i.e., PHA and NAC/PHA. Data, means ± SDs (*n* = 3 independent experiments). Experiments without the same top-labeled letters indicate significant changes according to multiple ANOVA comparisons (*p* < 0.05 to 0.0001).

The changes in the MitoSOX generation patterns with different concentrations and exposure times of PHA-treated breast cancer cells were detected by flow cytometry (Figure 5A,C). Following PHA treatment, the breast cancer cells exhibited concentration- and time-course-dependent overexpression for MitoSOX (+) (%) (Figure 5B,D).

To validate the role of ROS in the MitoSOX generation, NAC was used to pretreat PHA-treated breast cancer cells. The pretreatments with NAC suppressed PHA-induced MitoSOX overexpression in the breast cancer cells (Figure 5D).

### 3.6. PHA Triggered MitoMP Destruction in Breast Cancer Cells

**Figure 6:**
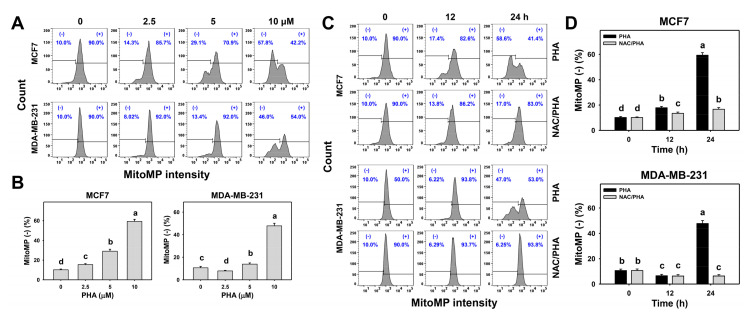
PHA triggered MitoMP depletion in two breast cancer cell lines. (**A**,**B**) MitoMP patterns and statistical analysis for concentration responses to PHA. Cells were incubated with PHA (0 (0.05% DMSO as control), 2.5, 5, and 10 μM) for 24 h. (−) indicates MitoMP (−) (%). (**C**,**D**) Patterns and statistical analysis of NAC pretreatment responses in MitoMP changes in PHA-post-treated breast cancer cells. Following pretreatment with NAC, cells were processed with post-treatment for 0 (control with 0.05% DMSO) and 10 μM PHA for 0, 12, and 24 h, i.e., PHA and NAC/PHA. Data, means ± SDs (*n* = 3 independent experiments). Experiments without the same top-labeled letters indicate significant changes according to multiple comparisons (*p* < 0.005 to 0.0001).

The changes in the MitoMP patterns due to different concentrations and exposure times of PHA-treated breast cancer cells were detected by flow cytometry (Figure 6A,C). PHA induced concentration- and time-course-dependent increases for MitoMP (−) (%) in the breast cancer cells (Figure 6B,D).

To validate the role of ROS in the MitoMP destruction, NAC was used to pretreat PHA-treated breast cancer cells. The pretreatment with NAC suppressed the PHA-induced MitoMP destruction in breast cancer cells (Figure 6D).

### 3.7. PHA Induced Antioxidant Signaling—GSR mRNA Expression—In Breast Cancer Cells

**Figure 7:**
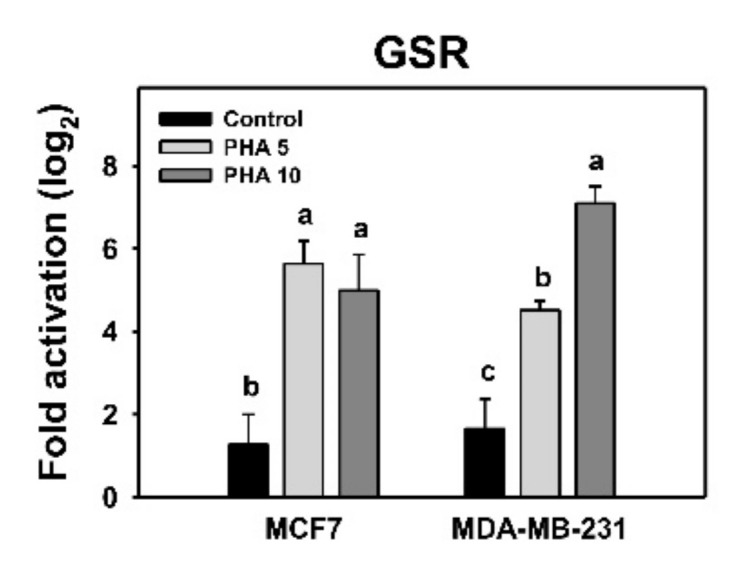
PHA induced *GSR* mRNA expression in breast cancer cells. Cells were incubated with PHA (0 (0.05% DMSO as control), 5 (PHA 5), and 10 (PHA 10) μM) for 24 h. Data, means ± SDs (*n* = 3 independent experiments). Experiments without the same top-labeled letters indicate significant changes according to multiple comparisons (*p* < 0.005 to 0.0001).

The *GSR* mRNA expression for two PHA-treated breast cancer cell lines was examined by qRT-PCR analysis. Following PHA treatment, the *GSR* mRNA was overexpressed in the breast cancer cells compared to control (Figure 7).

### 3.8. PHA Triggered γH2AX Expression in Breast Cancer Cells

**Figure 8:**
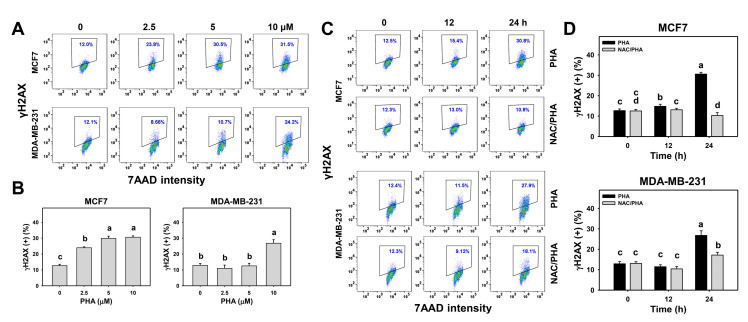
PHA triggered γH2AX overexpression in two breast cancer cell lines. (**A**,**B**) γH2AX patterns and statistical analysis for concentration responses of PHA. Cells were incubated with PHA (0 (0.05% DMSO as control), 2.5, 5, and 10 μM) for 24 h. Small box indicates γH2AX (+) (%). (**C**,**D**) Patterns and statistical analysis for NAC pretreatment responses in γH2AX intensity changes in PHA-post-treated breast cancer cells. Following pretreatment with NAC, cells were processed with post-treatment for 0 (control with 0.05% DMSO) and 10 μM PHA for 0, 12, and 24 h, i.e., PHA and NAC/PHA. Data, means ± SDs (*n* = 3 independent experiments). Experiments without the same top-labeled letters indicate significant changes according to multiple comparisons (*p* < 0.05 to 0.0001).

The changes in the γH2AX expression patterns with different concentrations and exposure times of PHA-treated breast cancer cells were detected by flow cytometry (Figure 8A,C). PHA induced concentration- and time-course-dependent increases for γH2AX (+) (%) in the breast cancer cells (Figure 8B,D).

To validate the role of ROS in the γH2AX expression, NAC was used to pretreat PHA-treated breast cancer cells. The pretreatment with NAC suppressed the PHA-induced γH2AX overexpression in the breast cancer cells (Figure 8D).

## 4. Discussion

PHA, one of the *P. peruviana*-derived natural products, has rarely been investigated, especially for its anticancer effect. In the current study, the antiproliferative, cell-cycle-disturbing, oxidative-stress-inducing, and DNA-damaging effects of PHA were validated in dose-dependence and time-course experiments with breast cancer cells. The detailed drug-acting mechanisms of PHA-induced anti-breast-cancer effects are discussed as follows.

### 4.1. PHA Is a Potential Antiproliferative Natural Product for Breast Cancer Cells

Recently, PHA was reported to provide antiproliferative effects against prostate (LNCaP) and renal (ACHN) cancer cells but induce less damage to human foreskin fibroblasts (HEF) [22], i.e., the IC_50_ concentrations at 72 h in an MTS assay were 0.11, 1.0, and >2 μM, respectively. However, these studies focused on the structure–activity relationship and reported the IC_50_ values of PHA without investigating the detailed mechanisms.

Breast cancer cells are reported to have several subtypes that respond differently to chemotherapy [8]. In the current study, the IC_50_ concentrations for PHA at 24 h in the ATP study for the three breast cancer cell lines ranged from 3.12 to 6.15 μM (Figure 1A), suggesting that PHA has cell-killing effects against different kinds of breast cancer cells, including those classified [3] as luminal A positive, HER2 positive, and Claudin-low (TNBC). The examination of possible killing effects on other types of breast cancer cells such as luminal B and basal are warranted in the future. Moreover, PHA may have the potential for combinatorial treatment with radiation and immunologically active compounds in a targeted therapy due to the immunoprofile characteristics of MCF7 (ER+, PR+/−, HER2−) and SKBR3 (ER−, PR−, HER2+) cells.

To provide a comparison with the clinically used anticancer drug cisplatin, the IC_50_ value for cisplatin at 24 h in the MTS assay for SKBR3 cells was 49.8 μM [34]. The IC_50_ concentrations for 48 h cisplatin in the ATP assay were 4.9, 17.9, and 26.9 μM for SKBR3, MCF7, and MDA-MB-231 cells, respectively [35]. For comparison, the IC_50_ concentrations for 24 h PHA in the ATP assay were 4.18, 3.12, and 6.15 μM for SKBR3, MCF7, and MDA-MB-231 cells, respectively (Figure 1A). Therefore, PHA has a higher potency to inhibit the proliferation of breast cancer cells than cisplatin. Although PHA exhibits low cell cytotoxicity to normal foreskin fibroblasts [22], it warrants a survival comparison of PHA with cisplatin in relation to more normal cell lines. The potential therapeutic index of PHA in terms of selectivity towards cancer cells needs to be examined in the future.

### 4.2. PHA Generates Oxidative Stress in Breast Cancer Cells

ROS-modulating strategies are commonly used for anticancer drug development [13,36,37]. ATP-production ability is proportional to mitochondrial function. When mitochondria show dysfunction, MitoSOX generation may show substantial upregulation. For example, manoalide induces ATP depletion, associated with MitoSOX generation and cell death in oral cancer cells [38]. PHA induces ATP depletion (Figure 1) and triggers oxidative-stress responses, including ROS and MitoSOX overexpression (Figure 4 and Figure 5), as well as MitoMP depolarization (Figure 6), in breast cancer cells. Accordingly, PHA is a ROS-modulating natural product with a substantial anti-breast-cancer effect.

Cellular redox homeostasis is regulated by both oxidative stress and antioxidant machinery. Cellular antioxidant machinery shows either activation or inactivation responses to oxidative-stress environments [39]. For example, a transient oxidative stress may induce a ROS detoxification response by activating superoxide dismutase (SOD) or catalase (CAT) [40]. By contrast, sustained oxidative stress may induce cancer cell death [40]. Culturing oocytes under a high-O_2_ condition induces *GSR*, glutathione peroxidase 1 (*GPX1*), *CAT*, *SOD1*, and *SOD2* mRNA overexpression [41]. Exogenous C8-ceramide induces ROS and apoptosis in lung cancer H1299 cells by upregulating mitochondrion-located SOD2 [42]. Similarly, PHA treatments for two breast cancer cell lines induce the *GSR* mRNA expression (Figure 7) associated with oxidative stress. It is possible that the *GSR* gene is activated in response to PHA-induced oxidative stress, but its ROS-scavenging capacity fails to counteract the high induction of oxidative stress in the end.

Some natural products generate ROS by themselves, such as naphthoquinones. For example, β-lapachone, a naphthoquinone derived from the lapacho tree’s bark, is a redox recycler [43] and a ROS-generating chemical [44]. β-lapachone can generate ROS by itself, oxidizing catalytic cysteine’s thiol group to the sulfinic acid form [43]. Whether PHA itself is a ROS-generating molecule remains unclear. This warrants the investigation of the ROS-generating ability of PHA in the future.

### 4.3. PHA Induces G2/M-Phase Arrest, Apoptotic Change, and γH2AX-Detected DNA Damage in Breast Cancer Cells

By activating cell-cycle checkpoints, the proliferation of cancer cells can be inhibited [45]. Several G2/M-arresting drugs have been developed to inhibit cancer cell proliferation, such as withaferin A in glioblastoma cells [46], sinularin in oral and breast cancer cells [27,47], genistein in colon cancer cells [48], and pevonedistat in breast cancer cells [49]. Similarly, PHA induced oxidative stress and G2/M arrest and resulted in apoptosis (according to flow cytometry and Western blotting) in breast cancer cells in spite of the low population of subG1 cells (Figure 2 and Figure 3).

SubG1 accumulation is not essential for apoptosis. Drugs may induce G2/M-phase arrest and trigger apoptosis but induce no subG1 accumulation with 24 h treatments, such as withametelin treatment for lung cancer A549 cells [50], (−)-anonaine treatment for lung cancer H1299 cells, and sinularin treatment for oral cancer Ca9-22 cells [47]. When cells arrested in G2/M phases become apoptotic, their DNA contents may degrade, and they may shift from G2/M to S or G1 phases without subG1 movement. In some cases, drugs with longer exposure may induce greater apoptosis compared to short exposure [47,51,52]. For example, subG1 accumulation was weak at 24 and 48 h of treatment but moderate at 72 h of treatment with the chalcone derivative Ch1 [52] and (−)-anonaine [47].

Several ROS-modulating drugs [30,38,53] were reported to activate both extrinsic (Cas-8) and intrinsic (Cas-9) apoptotic pathways; both pathways trigger apoptosis through the cleavage of downstream signaling molecules such as Cas-3. Similarly, PHA activates, in a similar manner, Cas-9, -8, and -3 in breast cancer cells, suggesting that PHA induces generic oxidative stress that activates apoptotic caspases.

After 24 h of treatment with 10 μM PHA, the various breast cancer cells showed strong cytotoxicity (about 80%) (Figure 1A); however, about 40% apoptosis was detected (Figure 3B). This suggests that PHA also induces another type of cell death, such as necrosis, which was detectable for 20% in terms of annexin V (−)/7AAD (+) analysis [28,29] (Figure 3B). Therefore, PHA induces more apoptosis than necrosis in breast cancer cells. Knowing the amount of necrosis allows discriminating nonspecific anticancer effects. It warrants a detailed investigation exploring the role of necrosis in PHA cytotoxicity to breast cancer cells in the future.

Moreover, oxidative stress is also a DNA-damage-inducing factor [24,54,55]. This was supported by our finding that PHA induced oxidative-stress responses such as ROS/MitoSOX overproduction and MitoMP depletion. Therefore, it caused DNA damage in breast cancer cells as detected by γH2AX (Figure 8).

### 4.4. NAC Suppresses PHA-Induced Antiproliferative Effects and ROS-Associated Changes in Breast Cancer Cells

All the PHA-induced changes such as antiproliferative effects, oxidative-stress induction, apoptosis, and DNA damage were recovered by NAC pretreatment. This held for ATP depletion, ROS/MitoSOX overproduction, MitoMP depletion, annexin V- and Western-blot-detected extrinsic and intrinsic apoptosis, and γH2AX-detected DNA damage. Accordingly, the PHA-induced antiproliferative effects, apoptosis, and DNA damage were mediated by oxidative-stress induction in the breast cancer cells.

## 5. Conclusions

Many bioactive compounds belonging to the withanolides isolated from several *Physalis* species show anticancer effects, but the action mechanisms have rarely been investigated. In the current study, we showed that PHA inhibits the proliferation of three kinds of breast cancer cells: MCF7 (ER+, PR+/−, HER2−), SKBR3 (ER−, PR−, HER2+), and MDA-MB-231 (TNBC). This antiproliferative effect of PHA against the breast cancer cells was proven to be oxidative-stress-dependent by NAC pretreatment. The cell-killing mechanisms of PHA include cell-cycle G2/M arrest, oxidative-stress induction, and DNA damage. Both the apoptosis and DNA damage were proven to be oxidative-stress-dependent. Therefore, PHA represents a potential anti-breast-cancer natural product, and its cell-killing mechanism is associated with the modulation of the oxidative-stress response, cell-cycle disturbance, apoptosis, and DNA damage.

## Data Availability

Data is contained within the article.
